# Contact with HIV prevention services highest in gay and bisexual men at greatest risk: cross-sectional survey in Scotland

**DOI:** 10.1186/1471-2458-10-798

**Published:** 2010-12-31

**Authors:** Lisa M McDaid, Graham J Hart

**Affiliations:** 1 MRC/CSO Social and Public Health Sciences Unit, 4 Lilybank Gardens, Glasgow, G12 8RZ, UK; 2 Centre for Sexual Health and HIV Research, Research Department of Infection and Population Health, University College London, Mortimer Market Centre, off Capper Street, London, WC1E 6JB, UK

## Abstract

**Background:**

Men who have sex with men (MSM) remain the group most at risk of acquiring HIV in the UK and new HIV prevention strategies are needed. In this paper, we examine what contact MSM currently have with HIV prevention activities and assess the extent to which these could be utilised further.

**Methods:**

Anonymous, self-complete questionnaires and Orasure™ oral fluid collection kits were distributed to men visiting the commercial gay scenes in Glasgow and Edinburgh in April/May 2008. 1508 men completed questionnaires (70.5% response rate) and 1277 provided oral fluid samples (59.7% response rate); 1318 men were eligible for inclusion in the analyses.

**Results:**

82.5% reported some contact with HIV prevention activities in the past 12 months, 73.1% obtained free condoms from a gay venue or the Internet, 51.1% reported accessing sexual health information (from either leaflets in gay venues or via the Internet), 13.5% reported talking to an outreach worker and 8.0% reported participating in counselling on sexual health or HIV prevention. Contact with HIV prevention activities was associated with frequency of gay scene use and either HIV or other STI testing in the past 12 months, but not with sexual risk behaviours. Utilising counselling was also more likely among men who reported having had an STI in the past 12 months and HIV-positive men.

**Conclusions:**

Men at highest risk, and those likely to be in contact with sexual health services, are those who report most contact with a range of current HIV prevention activities. Offering combination prevention, including outreach by peer health workers, increased uptake of sexual health services delivering behavioural and biomedical interventions, and supported by social marketing to ensure continued community engagement and support, could be the way forward. Focused investment in the needs of those at highest risk, including those diagnosed HIV-positive, may generate a prevention dividend in the long term.

## Background

Men who have sex with men (MSM) remain the group most at risk of acquiring HIV in the UK, accounting for 38% of diagnoses in 2008, 83% of which were probably acquired in the UK [1]. New HIV prevention strategies are needed and current policy initiatives set prevention as key to efforts to combat the HIV epidemic among MSM [1,2].

Combination prevention, which incorporates biomedical and behavioural, as well as social and structural, interventions has been argued as the way forward [3]. Furthermore, early, successful HIV prevention among MSM has been credited to the collective response of gay communities and their widespread adoption of safer sex behaviours, leading to calls for a renewed community response [4]. However, recent research has highlighted the evolving nature of the 'gay community' and its changing role in HIV prevention [5-7]; the divergence of different gay communities, and particularly ambivalence and changing community norms around safer sex [6]; the individualization of risk practices and responsibilities [5]; and structural, environmental and other physical changes in gay communities [7]. As such, the community's role in HIV prevention has changed and it has been argued that community-level prevention efforts have been difficult to maintain over the course of the epidemic as the threat of HIV has diminished, questioning the likelihood and potential of a renewed community response [8]. While further research is required to explore the meaning of 'gay community' to MSM in the UK, there is also a need to examine what contact MSM currently have with HIV prevention activities and to assess the extent to which these could be utilised in new prevention efforts.

Since 1996, we have surveyed the HIV-related sexual behaviour of MSM in Scotland [9-17]. This paper examines the extent to which MSM engage with existing HIV prevention activities, the factors associated with this, and discusses the opportunities presented for further intervention efforts.

## Methods

The 2008 MRC Gay Men's Survey collected anonymous, self-complete questionnaires and (Orasure™) oral fluid specimens. Representative samples were recruited from commercial gay venues (12 bars and 2 saunas) in Glasgow and Edinburgh, Scotland's two largest cities, using time and location sampling [15]. 1514 men participated in the survey (70.8% response rate [RR]); 1508 completed questionnaires (70.5% RR) and 1277 provided oral fluid samples (59.7% RR). Of the 1508 men who completed questionnaires, 54 (3.6%) heterosexual men reported no sexual contact with men in the previous 12 months and are excluded from the sample.

1318 men provided data on the current HIV prevention variables and 136 men with missing data on any of these are excluded from the analyses in this paper. When compared in multivariate analysis, men who provided data on the HIV prevention variables had lower odds of being surveyed in Glasgow (AOR = 0.52, 95% CI 0.33-0.81), being surveyed in a sauna (AOR = 0.43, 95% CI 0.22-0.85), and of having had an STI in the past 12 months (AOR = 0.46, 95% CI 0.25-0.79) than men with missing data on these variables.

Questionnaires included demographics, HIV testing history and sexual risk behaviour in the past 12 months [Additional file 1]. Participants were asked if they had, in the past 12 months, picked up sexual health leaflets in bars, clubs or saunas; looked for safer sex/sexual health information on the Internet; obtained free condoms from bars, clubs, saunas or the Internet; talked to an outreach worker in a bar, club or sauna; or participated in one to one or group counselling sessions on sexual health or HIV prevention.

Oral fluid specimens were analysed at the West of Scotland Specialist Virology Centre (screened for anti-HIV using an enzyme immunoassay; positives re-screened, and repeat reactives confirmed using Western Blot). Data were analysed with SPSS 15.0. Logistic regression was used to estimate odds ratios and 95% confidence intervals (CI). Ethical approval was granted by University of Glasgow, Faculty of Medicine Ethics Committee.

## Results

### Sample Characteristics

Sample characteristics are shown in Table 1. The majority were surveyed in bars and identified as gay. The majority were aged over 26 years; 76.8% lived in the Glasgow or Edinburgh areas. Only 3.3% reported being from minority ethnic groups. Just less than half reported degree or post-graduate education and the majority were employed. Many respondents (46.8%) visited the gay scene at least once a week. Just under half had been tested for HIV or other sexually transmitted infections (STIs) in the past 12 months; 3.5% had a HIV-positive oral fluid sample. Most men (96.2%) reported some sexual contact in the past 12 months, 12.4% reported UAI with 2+ partners, 20.5% reported UAI with casual partners, 24.9% reported UAI with partners of unknown/discordant HIV status, and 8.3% had had an STI in the past 12 months (Table 1).

**Table 1:** Sample characteristics (N = 1318)

	n	%
***Demographics***		
**Survey location**		
Edinburgh	559	42.2
Glasgow	759	57.6
**Survey venue**		
Bar	1243	94.3
Sauna	75	5.7
**Sexual orientation**		
Gay	1180	90.1
Bisexual	113	8.6
Straight	17	1.3
**Age**		
16-25 years	378	29.0
26+ years	926	71.0
**Area of residence**		
Glasgow	538	41.9
Edinburgh	449	34.9
Rest of Scotland	204	15.9
Rest of UK	67	5.2
Overseas	27	2.1
**Ethnicity**		
White (UK, Irish or other)	1272	96.7
Minority ethnic group*	44	3.3
**Qualifications**		
Secondary	195	15.9
Further/vocational	460	37.5
Degree/post-graduate	571	46.6
**Frequency of gay scene use**		
Once month or less	327	25.5
2/3 times a month	354	27.7
1/2 times a week	432	33.8
4/5 times a week	167	13.0
**HIV treatment optimism 1†**		
Disagree	938	74.2
Agree	327	25.8
**HIV treatment optimism 2†**		
Disagree	1056	83.5
Agree	209	16.5
***Sexual Health***		
**HIV test in the past 12 months**		
No	667	51.9
Yes	618	48.1
**Other sexually transmitted infection (STI) test in the past 12 months**		
No	711	54.3
Yes	599	45.7
**HIV status (oral fluid specimen result)**		
HIV-negative	1077	81.9
HIV-positive	46	3.5
Did not provide oral fluid specimen‡	192	14.6
***Sexual Risk Behaviour in the past 12 months***		
**Number of sexual partners**		
Less than 10	927	72.7
10 or more	348	27.3
**Number of anal sex partners**		
Less than 10	1126	89.3
10 or more	135	10.7
**Number of unprotected anal intercourse (UAI) partners**		
0/1 partner	1127	87.6
2 or more partners	160	12.4
**UAI with casual partners**		
No	1023	79.5
Yes	264	20.5
**UAI with partners of unknown/discordant HIV status**		
No	966	75.1
Yes	321	24.9
**STI**		
No	1202	91.7
Yes	109	8.3

*Black African, Black Caribbean, Indian, Pakistani, Chinese, Arab, Latin American & Other/Mixed.

† HIV treatment optimism 1 - 'I am less worried about HIV infection now that treatments have improved', HIV treatment optimism 2 - 'I believe that new drug therapies make people with HIV less infectious'.

‡ An additional 4 samples were not returned from the laboratory.

### Contact with HIV prevention activities

Overall, 1135 men (82.5%) reported some contact with HIV prevention activities in the past 12 months (Table 2). Having obtained free condoms from a gay venue or the Internet was the most frequently reported HIV prevention activity (73.1%), but over a third of men also reported picking up sexual health leaflets in gay venues or using the Internet to look for safer sex or sexual health information. Overall, 674 men (51.1%) reported accessing sexual health information (from either leaflets in bars, clubs or saunas, or via the Internet). Only 13.5% reported talking to an outreach worker and only 8.0% reported participating in one to one or group counselling on sexual health or HIV prevention. Only 47 men (3.6%) reported use of all four HIV prevention activities.

**Table 2:** Contact with HIV prevention activities in the past 12 months (N = 1318)

	Contact in past 12 months			
	
	Yes		No	
	
	n	%	n	%
**Contact with HIV prevention activities in last 12 months**				
Any	1082	82.1	236	17.9
Obtained free condoms from a bar/club/sauna or the Internet	964	73.1	354	26.9
Picked up sexual health leaflet in a bar/club/sauna	508	38.6	810	61.5
Looked for safer sex/sexual health information on the Internet	459	34.8	859	65.2
Talked to an outreach worker in a bar/club/sauna	178	13.5	1140	86.5
Participated in one to one or group counselling on sexual health or HIV prevention	105	8.0	1213	92.0

### Factors associated with contact with HIV prevention activities

Table 3 shows the factors associated with contact with HIV prevention activities. The likelihood of engaging with all of the listed HIV prevention activities was higher among men who were more frequent scene users, men who had had an HIV, or other STI, test in the past 12 months, men who reported 10 or more sexual partners in the past 12 months, and men who reported having had an STI in the past 12 months (it should be noted that some STIs can be transmitted during lower risk sexual practices so are not necessarily exclusively indicators of high risk behaviour). The likelihood of obtaining free condoms was lower among men who identified as straight, who were aged 26+ years or who resided in areas of the UK other than Scotland. The likelihood of accessing sexual health information via gay venues or the Internet (which were combined for these analyses) was higher among men surveyed in saunas, men from minority ethnic groups, men with further and higher educational qualifications, but lower among men who did not provide oral fluid samples. The likelihood of talking to an outreach worker was lower among men aged 26+ years and men who lived in the rest of Scotland (compared with Glasgow). The likelihood of participating in one to one or group counselling was higher among men who agreed with the HIV treatment optimism statement, 'I believe that new drug therapies make people with HIV less infectious'. The likelihood of accessing sexual health information or participating in counselling was higher among HIV-positive than HIV-negative men, but there were no differences in the proportions reporting obtaining free condoms or talking to an outreach worker, or between diagnosed and undiagnosed HIV-positive men. Almost exclusively, the likelihood of contact with HIV prevention activities was higher among men who reported more sexual partners and any of the sexual risk behaviours. A notable exception was UAI with partners of unknown/discordant HIV status, which was not associated with any HIV prevention activities.

**Table 3:** Factors associated with contact with HIV prevention activities in the past 12 months (N = 1318)

	Obtained free condoms from a bar/club/sauna or the Internet			Picked up sexual health leaflet in a bar/club/sauna or looked for safer sex/sexual health information on the Internet			Talked to an outreach worker in a bar/club/sauna			Participated in one to one or group counselling on sexual health or HIV prevention		
	
	n	%	OR (95% CI)	n	%	OR (95% CI)	n	%	OR (95% CI)	n	%	OR (95% CI)
***Sample characteristics***												
**Survey location**												
Edinburgh	414	74.1	1	296	53.0	1	73	13.1	1	47	8.4	1
Glasgow	550	72.5	0.92 (0.72-1.18)	378	49.8	0.88 (0.71-1.10)	105	13.8	1.07 (0.78-1.47)	58	7.6	0.90 (0.60-1.35)
**Survey venue**												
Bar	910	73.2	1	627	50.4	1	168	13.5	1	101	8.1	1
Sauna	54	72.0	0.94 (0.56-1.58)	47	62.7	*1.65* *(1.02-2.67)*	10	13.3	0.98 (0.50-1.95)	4	5.3	0.64 (0.23-1.78)
**Sexual orientation**												
Gay	873	74.0	1	604	51.2	1	161	13.6	1	90	7.6	1
Bisexual	76	67.3	0.72 (0.48-1.09)	60	53.1	1.08 (0.73-1.59)	15	13.3	0.97 (0.55-1.71)	13	11.5	1.57 (0.85-2.92)
Straight	8	47.1	*0.31* *(0.12-0.82)*	5	29.4	0.40 (0.14-1.14)	0	0	-	1	5.9	0.76 (0.10-5.77)
**Age**												
16-25 years	299	79.1	1	198	52.4	1	65	17.2	1	38	10.1	1
26+ years	655	70.7	*0.64* *(0.48-0.85)*	469	50.6	0.93 (0.73-1.19)	111	12.0	*0.66* *(0.47-0.92)*	65	7.0	0.68 (0.44-1.03)
**Area of residence**												
Glasgow	400	74.3	1	280	52.0	1	85	15.8	1	47	8.7	1
Edinburgh	341	75.9	1.09 (0.82-1.46)	238	53.0	1.04 (0.81-1.34)	60	13.4	0.82 (0.58-1.18)	37	8.2	0.94 (0.60-1.47)
Rest of Scotland	141	69.1	0.77 (0.54-1.10)	104	51.0	0.96 (0.69-1.32)	20	9.8	*0.58* *(0.35-0.97)*	10	4.9	0.54 (0.27-1.09)
Rest of UK	42	62.7	*0.58* *(0.34-0.99)*	28	41.8	0.66 (0.40-1.11)	6	9.0	0.52 (0.22-1.25)	7	10.4	1.22 (0.53-2.82)
Overseas	21	77.8	1.21 (0.48-3.05)	12	44.4	0.74 (0.34-1.60)	2	7.4	0.43 (0.10-1.83)	1	3.7	0.40 (0.05-3.03)
**Ethnicity**												
White (UK, Irish or other)	933	73.3	1	639	50.2	1	168	13.2	1	101	7.9	1
Minority ethnic group*	29	65.9	0.70 (0.37-1.33)	33	75.0	*2.97* *(1.49-5.93)*	9	20.5	1.69 (0.80-3.58)	3	6.8	0.85 (0.26-2.79)
**Qualifications**												
Secondary	146	74.9	1	85	43.6	1	24	12.3	1	17	8.7	1
Further/vocational	337	73.3	0.92 (0.63-1.35)	243	52.8	*1.45* *(1.03-2.03)*	63	13.7	1.13 (0.68-1.87)	30	6.5	0.73 (0.39-1.36)
Degree/post-graduate	420	73.6	0.93 (0.64-1.36)	306	53.6	*1.49* *(1.08-2.07)*	78	13.7	1.13 (0.69-1.84)	42	7.4	0.83 (0.46-1.50)
**Frequency of gay scene use**												
Once month or less	197	60.2	1	139	42.5	1	30	9.2	1	18	5.5	1
2/3 times a month	261	73.7	*1.85* *(1.34-2.56)*	182	51.4	*1.43* *(1.06-1.94)*	36	10.2	1.12 (0.67-1.87)	24	6.8	1.25 (0.67-2.35)
1/2 times a week	338	78.2	*2.37* *(1.73-3.26)*	245	56.7	*1.77* *(1.33-2.37)*	62	14.4	*1.66* *(1.05-2.63)*	30	6.9	1.28 (0.70-2.34)
4/5 times a week	144	86.2	*4.13* *(2.52-6.76)*	95	56.9	*1.79* *(1.23-2.60)*	41	24.6	*3.22* *(1.93-5.39)*	27	16.2	*3.31* *(1.77-6.21)*
**HIV treatment optimism 1**†												
Disagree	696	74.2	1	497	53.0	1	123	13.1	1	73	7.8	1
Agree	236	72.2	0.90 (0.68-1.20)	153	46.8	0.78 (0.61-1.00)	46	14.1	1.09 (0.75-1.56)	28	8.6	1.11 (0.70-1.75)
**HIV treatment optimism 2**†												
Disagree	778	73.7	1	545	51.6	1	138	13.1	1	72	6.8	1
Agree	154	73.7	1.00 (0.71-1.40)	105	50.2	0.95 (0.70-1.27)	31	14.8	1.16 (0.76-1.77)	29	13.9	*2.20* *(1.39-3.49)*
***Sexual Health***												
**HIV test in the past 12 months**												
No	460	69.0	1	296	44.4	1	52	7.8	1	23	3.4	1
Yes	486	78.6	*1.66* *(1.29-2.13)*	366	59.2	*1.82* *(1.46-2.27)*	121	19.6	*2.88* *(2.04-4.07)*	79	12.8	*4.10* *(2.54-6.62)*
**Other STI test in the past 12 months**												
No	470	66.1	1	313	44.0	1	57	8.0	1	32	4.5	1
Yes	489	81.6	*2.28* *(1.76-2.95)*	357	59.6	*1.88* *(1.51-2.34)*	119	19.9	*2.85* *(2.03-3.98)*	70	11.7	*2.81* *(1.82-4.33)*
**HIV status (oral fluid specimen result)**												
HIV-negative	787	73.1	1	558	51.8	1	149	13.8	1	82	7.6	1
HIV-positive	35	76.1	1.17 (0.59-2.34)	32	69.6	*2.13* *(1.12-4.03)*	7	15.2	1.12 (0.49-2.55)	11	23.9	*3.81* *(1.87-7.79)*
Did not provide oral fluid specimen	139	72.4	0.97 (0.69-1.36)	82	42.7	*0.69* *(0.51-0.95)*	22	11.5	0.81 (0.50-1.30)	12	6.3	0.81 (0.43-1.51)
***Sexual Risk Behaviours in past 12 months***												
**Number of sexual partners**												
Less than 10	644	69.5	1	450	48.5	1	111	12.0	1	66	7.1	1
10 or more	296	85.1	*2.50* *(1.81-3.47)*	211	60.6	*1.63* *(1.27-2.10)*	62	17.8	*1.59* *(1.14-2.24)*	37	10.6	*1.55* *(1.02-2.37)*
**Number of anal intercourse partners**												
Less than 10	813	72.2	1	568	50.4	1	139	12.3	1	86	7.6	1
10 or more	122	90.4	*3.61* *(2.01-6.50)*	88	65.2	*1.84* *(1.27-2.67)*	32	23.7	*2.21* *(1.43-3.41)*	16	11.9	1.63 (0.92-2.87)
**Number of unprotected anal intercourse (UAI) partners**												
0/1 partner	816	72.4	1	576	51.1	1	142	12.6	1	83	7.4	1
2 or more partners	135	84.4	*2.06* *(1.32-3.22)*	90	56.3	1.23 (0.88-1.72)	34	21.3	*1.87* *(1.23-2.84)*	22	13.8	*2.01* *(1.21-3.31)*
**UAI with casual partners**												
No	737	72.0	1	513	50.1	1	129	12.6	1	79	7.7	1
Yes	214	81.1	*1.66* *(1.19-2.33)*	153	58.0	*1.37* *(1.04-1.80)*	47	17.8	*1.50* *(1.04-2.16)*	26	9.8	1.31 (0.82-2.08)
**UAI with partners of unknown/discordant HIV status**												
No	701	72.6	1	503	52.1	1	127	13.1	1	74	7.7	1
Yes	250	77.9	1.33 (0.99-1.80)	163	50.8	0.95 (0.74-1.22)	49	15.3	1.19 (0.83-1.70)	31	9.7	1.29 (0.83-2.00)
**STI**												
No	870	72.4	1	593	49.3	1	148	12.3	1	77	6.4	1
Yes	89	81.7	*1.70* *(1.03-2.80)*	76	69.7	*2.37* *(1.55-3.61)*	26	23.9	*2.23* *(1.39-3.58)*	24	22.0	*4.13* *(2.48-6.86)*

OR = odds ratio; 95% CI = 95% confidence interval; significant odds ratios are shown in italics, p < 0.05.

*Black African, Black Caribbean, Indian, Pakistani, Chinese, Arab, Latin American & Other/Mixed.

† HIV treatment optimism 1 - 'I am less worried about HIV infection now that treatments have improved', HIV treatment optimism 2 - 'I believe that new drug therapies make people with HIV less infectious'.

Factors significant at the bivariate level were entered into a multivariate model for each of the HIV prevention activities (Table 4). The factors that remained significantly associated with obtaining free condoms were: age, frequency of gay scene use, STI testing in the past 12 months, and having 10 or more sexual partners in the past 12 months. The factors associated with accessing sexual health information were ethnicity and frequency of gay scene use, while odds remained lower among men who did not provide oral fluid specimens. Factors associated with talking to an outreach worker were frequency of gay scene use, and HIV and STI testing. Factors associated with participating in counselling were frequency of gay scene use, HIV treatment optimism, HIV testing, HIV-positive status and having had an STI in the past 12 months.

**Table 4:** Factors associated with contact with HIV prevention activities in the past 12 months: multivariate logistic regression (N = 1318)

	Obtained free condoms from a bar/club/sauna or the Internet		Picked up sexual health leaflet in a bar/club/sauna or looked for safer sex/sexual health information on the Internet		Talked to an outreach worker in a bar/club/sauna		Participated in one to one or group counselling on sexual health or HIV prevention	
	
	AOR	(95% CI)	AOR	(95% CI)	AOR	(95% CI)	AOR	(95% CI)
**Survey venue**								
Bar			1					
Sauna			1.46	(0.86-2.46)				
**Sexual orientation**								
Gay	1							
Bisexual	0.87	(0.56-1.35)						
Straight	1.03	(0.28-3.74)						
**Age**								
16-25 years	1				1			
26+ years	*0.73*	*(0.53-0.99)*			0.76	(0.53-1.09)		
**Area of residence**								
Glasgow	1				1			
Edinburgh	1.11	(0.81-1.51)			0.79	(0.54-1.15)		
Rest of Scotland	0.89	(0.61-1.30)			0.63	(0.37-1.09)		
Rest of UK	0.73	(0.42-1.29)			0.63	(0.26-1.56)		
Overseas	1.26	(0.46-3.46)			0.43	(0.10-1.94)		
**Ethnicity**								
White (UK, Irish or other)			1					
Minority ethnic group*			*2.91*	*(1.43-5.95)*				
**Qualifications**								
Secondary			1					
Further/vocational			1.35	(0.95-1.93)				
Degree/post-graduate			1.36	(0.96-1.93)				
**Frequency of gay scene use**								
Once month or less	1		1		1		1	
2/3 times a month	*1.67*	*(1.19-2.35)*	1.34	(0.97-1.84)	1.01	(0.60-1.71)	1.10	(0.57-2.13)
1/2 times a week	*1.92*	*(1.36-2.69)*	*1.62*	*(1.19-2.20)*	1.30	(0.80-2.11)	1.01	(0.54-1.92)
4/5 times a week	*3.13*	*(1.85-5.27)*	*1.51*	*(1.01-2.26)*	*2.16*	*(1.24-3.76)*	*2.43*	*(1.23-4.82)*
**HIV treatment optimism 2**†								
Disagree							1	
Agree							*2.19*	*(1.33-3.61)*
**HIV test in the past 12 months**								
No	1		1		1		1	
Yes	0.71	(0.49-1.03)	1.30	(0.95-1.79)	*1.75*	*(1.08-2.83)*	*3.44*	*(1.82-6.49)*
**Other STI test in the past 12 months**								
No	1		1		1		1	
Yes	*2.44*	*(1.67-3.57)*	1.31	(0.95-1.81)	*1.64*	*(1.01-2.64)*	0.99	(0.54-1.80)
**HIV status (oral fluid specimen result)**								
HIV-negative			1				1	
HIV-positive			1.88	(0.96-3.67)			*3.22*	*(1.42-7.29)*
Did not provide oral fluid specimen			*0.70*	*(0.50-0.96)*			0.82	(0.42-1.60)
**Number of sexual partners**								
Less than 10	1		1		1		1	
10 or more	*1.76*	*(1.19-2.62)*	1.16	(0.84-1.60)	0.98	(0.61-1.58)	0.97	(0.60-1.58)
**Number of anal intercourse partners**								
Less than 10	1		1		1			
10 or more	1.67	(0.83-3.35)	1.20	(0.75-1.91)	1.56	(0.85-2.85)		
**Number of unprotected anal intercourse (UAI) partners**								
0/1 partner	1				1		1	
2 or more partners	1.06	(0.60-1.88)			1.18	(0.66-2.11)	1.38	(0.80-2.41)
**UAI with casual partners**								
No	1		1		1			
Yes	1.16	(0.77-1.77)	1.15	(0.86-1.55)	1.10	(0.68-1.79)		
**STI**								
No	1		1		1		1	
Yes	0.73	(0.42-1.27)	1.54	(0.98-2.44)	1.35	(0.80-2.27)	*2.53*	*(1.40-4.57)*

AOR = adjusted odds ratio; 95% CI = 95% confidence interval; significant odds ratios are shown in italics, p < 0.05.

*Black African, Black Caribbean, Indian, Pakistani, Chinese, Arab, Latin American & Other/Mixed.

† HIV treatment optimism 2 - 'I believe that new drug therapies make people with HIV less infectious'.

## Discussion

In a study of the economic implications of HIV infection in the UK, it has been estimated that the lifetime HIV-related costs for diagnosed HIV-positive individuals is between £280,000 and £360,000 and preventing the HIV infections acquired and diagnosed in the UK in 2008 would save £1.1 billion in future HIV-related costs [1]. MSM, who accounted for over a third of diagnoses in 2008, most of which were probably acquired in the UK [1], are therefore a high priority for HIV prevention interventions. This is the first UK paper to report engagement with HIV prevention strategies in MSM since the advent of antiretroviral treatment in 1996.

The majority of the MSM in our community-based surveys reported having contact with HIV prevention activities in the past 12 months. Obtaining free condoms was reported by almost three quarters of the sample. Half also reported picking up sexual health leaflets in a bar, club or sauna or looking for safer sex or sexual health information on the Internet. Talking to outreach workers in gay venues or participating in one to one or group counselling on sexual health or HIV prevention was less common, reported by just one in ten respectively. Comparable data are not available elsewhere in the UK, but our findings are similar to those of the US National HIV Behavioural Surveillance survey of MSM, which found 80% had received free condoms, but few had participated in individual or group HIV prevention interventions in the past 12 months (15% and 8%, respectively) [18].

In multivariate analysis, each of the HIV prevention activities remained associated with frequency of gay scene use, but not with sexual risk behaviours (with the exception of having ten or more sexual partners, which itself only remained associated with obtaining free condoms). Frequency of gay scene use could be taken as a proxy for risk behaviour and the two were correlated, but there were no significant interactions between the two in any of the multivariate HIV prevention models and when separate models were fitted for each of the correlated variables and compared with the final models to check for the effects of collinearity, no substantively important differences were observed (data not shown). It is also of particular note that contact with HIV prevention activities (with the exception of accessing sexual health information) remained associated with either HIV or other STI testing in the past 12 months, with utilising counselling also more likely among men who reported having had an STI in the past 12 months and HIV-positive men.

There are some limitations to note when considering these results. Only men who visited the venues surveyed had the opportunity to participate and caution should be taken when generalising to a wider population of gay men. The data are cross-sectional and participants were only asked if they had contact with a pre-determined list of HIV prevention activities, not the extent of this contact, the quality, intensity, or frequency of exposure to these activities, or what, if any, impact it had on them. Other activities could have been available to, and utilised by, the men who participated in this survey. It is recognised that these factors should be assessed in the future to accurately assess the potential of prevention services to change risk behaviour. Furthermore, our examination of associations between prevention activities and sexual risk is limited to the behaviours for which data were collected and these aggregate measures may miss more complex risk reduction strategies being employed by individual men. However, the results provide interesting insight into men's current contact with a range of HIV prevention activities available to them and the following discussion concentrates on the implications of the findings for future HIV prevention efforts.

It is encouraging that the majority of men had some contact with HIV prevention activities. Condom provision is a core component of HIV prevention [19]. Considerably more men reported obtaining free condoms than any of the other activities and it presents an opportunity for further prevention efforts and community engagement (e.g. using existing condom distribution methods to distribute other sexual health or HIV communications). Half of the men surveyed had accessed such sexual health materials, either via leaflets or online, and mass communication, or social marketing, campaigns (some of which include the provision and distribution of leaflets in addition to more general poster or media advertising) continue to be frequently employed as a means of HIV-related health promotion [20]. Indeed, the recently published Scottish HIV Action Plan includes a specific recommendation to develop and implement social marketing materials for MSM [2]. With the Internet now a recognised setting that MSM are likely to utilise to meet sexual partners [21], and evidence supporting online health promotion [22], interest has grown in using the Internet as a setting for HIV prevention interventions. However, both mass communication and Internet interventions are often difficult to evaluate and, to date, have been shown to have limited impact on risk behaviour [20,23].

Talking to outreach workers in bars, clubs or saunas, though markedly less common than obtaining free condoms or accessing sexual information via leaflets or the Internet, appears to continue to have a role in increasing access to sexual health services, as evidenced here by the association between this and HIV/STI testing. Our previous evaluation of the *Gay Men's Task Force *peer education intervention reported similar findings, with higher rates of service use among men who had talked to peer educators in bars [10]. Outreach workers are ideally placed to direct at risk men to sexual health services and to support other, more general, social marketing campaigns.

Utilising one to one or group counselling on sexual health or HIV prevention, the most intensive form of prevention examined here, was also the least commonly reported. The association between testing, HIV, STIs and counselling could reflect the risk reduction counselling provided by sexual health practitioners when men present for testing or treatment. As such, these interactions represent the opportunity for interventions to effect behaviour change, as noted in existing UK guidance [24]. Intervention delivery by health care providers, and in settings where people receive routine HIV care or services, are two of the characteristics of successful interventions [25]. Sexual health services would be appropriate settings for such interventions in the UK. They would also be the most appropriate setting for the combination of these with biomedical interventions, such as early treatment for HIV to contribute to reducing community viral load, in which there is growing interest [26-29].

This is a highly sexually active sample, and at the bivariate level, contact with HIV prevention activities was consistently more common among men with more sexual partners and greater sexual risk behaviours (with the exception of UAI with partners of unknown or discordant HIV status). Although this suggested men at greater risk were making use of existing activities (which theoretically could help reduce their risk), it is striking that these associations did not remain significant in multivariate analysis (though, as noted above, frequency of gay scene use could be taken as a proxy for risk behaviour). Prevention efforts have been ongoing at a time when HIV-related sexual risk behaviour has essentially stabilised in this population (at a high level first observed in 2002 [13,30]). Although this plateau could be interpreted as evidence of successful prevention efforts, and stabilisation of risk behaviours during periods of intensified prevention efforts have been noted elsewhere [31,32], continued efforts are needed to address sexual risk among the minority of men for whom this appears to have become the norm [30]. Our results suggest high-risk men, those in contact with sexual health services, are accessing HIV prevention. Therefore, it is possible such prevention needs to be renewed, reinvigorated, or changed entirely, if reductions in sexual risk behaviours are to be achieved.

## Conclusions

Calls for a renewed community response to HIV among MSM have been based on the early, successful HIV prevention and the widespread adoption of safer sex behaviours, which resulted from this [4]. However, recent research has highlighted the evolving nature of the gay community [5-7], questioning the likelihood and potential of a renewed community response to the HIV epidemic among MSM. Furthermore, although there is growing evidence of the potential effectiveness of individual, group and community-level behavioural HIV prevention interventions in reducing high-risk sexual behaviour among MSM [33], behaviour change alone is unlikely to achieve the sustained reductions in HIV transmission necessary to change the course of the HIV epidemic [3]. Multi-level, combination prevention could be the way forward [3], and opportunities exist to exploit men's current contact with HIV prevention activities further.

We have demonstrated that men at highest risk, and those likely to be in contact with sexual health services, are those who report most contact with current HIV prevention activities. In addition, our previous research has shown that peer outreach can increase the uptake of sexual health services [10]. If we are to offer combination prevention, a model for delivering this could therefore include outreach by peer health workers, encouraging increased uptake of sexual health services delivering behavioural and biomedical interventions, and supported by social marketing to ensure continued community engagement and support. Whilst general education to encourage condom use by MSM should continue, focused investment in the needs of those at highest risk, including those diagnosed HIV positive, may generate a prevention dividend in the long term.

## Competing interests

The authors declare that they have no competing interests.

## Authors' contributions

LMcD devised the paper, conducted the analyses and wrote the first draft. GH made substantial comments and contributed to subsequent drafts. Both authors approved the final version of the manuscript submitted for publication.

## Pre-publication history

The pre-publication history for this paper can be accessed here:

http://www.biomedcentral.com/1471-2458/10/798/prepub

## Supplementary Material

Additional file 1 **Questionnaire**. 2008 survey questionnaire Click here for file
