# Supplementary material for: Contact with HIV prevention services highest in gay and bisexual men at greatest risk: cross-sectional survey in Scotland
Source: BMC Public Health. 2010 Dec 31;10:798. doi: 10.1186/1471-2458-10-798 (PMC3022866; doi:10.1186/1471-2458-10-798)
Supplement: Additional file 1 — Questionnaire. 2008 survey questionnaire [file 1471-2458-10-798-S1.DOC]

**24. Have you had a sexually transmitted infection in the last 12 months?**

***(please tick all that apply)***

Yes, gonorrhoea 🞏

Yes, chlamydia 🞏

Yes, syphilis 🞏

Yes, other 🞏

No 🞏

**25. Have you been tested for STIs in the last 12 months?**

***(please tick all that apply)***

Yes, at the Steve Retson Project 🞏

Yes, at another sexual health / GUM clinic 🞏

Yes, at the Brownlee 🞏

Yes, at a GP 🞏

Yes, in a gay bar/sauna 🞏

Yes, I used a home testing kit 🞏

No 🞏

26. In the last 12 months have you done any of the following?

Yes No

Picked up sexual health leaflet

in a bar/club/sauna 🞏 🞏

Looked for safer sex/sexual health

information on the Internet 🞏 🞏

Obtained free condoms from

a bar/club/sauna or the Internet 🞏 🞏

Talked to an outreach worker

in a bar/club or sauna 🞏 🞏

Participated in one to one or group

counselling sessions on sexual health

or HIV prevention 🞏 🞏

**P.T.O**

**27. We are looking for new ways to prevent HIV. Should the following research studies take place, which would you be willing to take part in?**

Don’t

Yes No know

Behaviour change programme

(e.g. support using condoms) 🞏 🞏 🞏

Circumcision

(surgical removal of foreskin) 🞏 🞏 🞏

Using rectal microbicides

(special lube to prevent HIV) 🞏 🞏 🞏

HIV vaccine 🞏 🞏 🞏

28. In the last month, which venues have you been to on the gay scene?

*(please tick all that apply)*

Bars 🞏

Clubs or club nights 🞏

Saunas 🞏

Cruising areas 🞏

Internet chat rooms (e.g. gaydar) 🞏

29. In the last month, how often did you go out on the gay scene?

4-5 times a week 🞏

1-2 times a week 🞏

2-3 times a month 🞏

once a month or less 🞏

Many thanks for your time and help in completing this questionnaire.

Please seal it in the envelope provided and return it to the researcher who gave it to you.

|  |  | **IDNO** |  | |
| --- | --- | --- | --- | --- |
|  |  |  |  |  |
|  |  |  |  |  |
|  |  |  |  |  |

###### survey

###### 2008

# gay & bisexual men’s sexual health

**Glasgow**

**Please tick or give a number as appropriate. Try to answer all the questions as fully as you can.**

**1. How would you describe your sexual orientation?**

Gay 🞏

Bisexual 🞏

Straight 🞏

Other 🞏

(please specify)____________________________

2. What age are you? __________

3. What is the first part of

your postcode? (e.g. G42)

4. Which of the following best describes you?

Black African 🞏 Chinese 🞏

Black Caribbean 🞏 White (UK) 🞏

Bangladeshi 🞏 White (Irish) 🞏

Indian 🞏 White (Other) 🞏

Pakistani 🞏

Other/Mixed (please specify)__________________

5. What nationality are you?

*(e.g. Scottish, Polish)*______________________

6. Are you currently…?

Employed 🞏 Self-employed 🞏

Unemployed 🞏 Retired 🞏

Student 🞏

**7. Could you tell us what your highest educational qualifications are? *(e.g. CSEs, Highers, Degree)***

**________________________________________**

**8. Are you currently in a relationship with a man?**

Yes 🞏 how long?_ ____years_____months

No 🞏

**9. With how many men have you had any sexual contact in the last 12 months?**

**10. With how many men have you had oral sex in the last 12 months?**

**11. With how many men have you had anal sex in the last 12 months?**

**If you haven't had anal sex in the last 12 months please go to question 17 🡻**

**12. With how many men have you had anal sex WITHOUT a condom in the last 12 months?**

**13. How often was this with a casual partner?**

Always 🞏 Sometimes 🞏 Never 🞏

**14. How often did you know these partners’ HIV status?**

Always 🞏 Sometimes 🞏 Never 🞏

**15. Were any of these partners HIV positive?**

Yes, all 🞏 Yes, some 🞏

No 🞏 Don’t know 🞏

**16. Were you…**

Always the active (top) partner 🞏

Mostly the active (top) partner 🞏

Equally both (versatile) 🞏

Mostly the passive (bottom) partner 🞏

Always the passive (bottom) partner 🞏

17. Circumcision is the surgical removal of the foreskin. Do you have a preference for a circumcised (cut) or uncircumcised (uncut) partner?

Circumcised 🞏 Uncircumcised 🞏

No preference 🞏

18. Are you circumcised? Yes 🞏 No 🞏

If yes, what age were you when

you were circumcised? __________

19. Do you believe the following to be true? Don’t Yes No know

Circumcision reduces risk of HIV 🞏 🞏 🞏

Circumcision reduces risk of

other STIs 🞏 🞏 🞏

Circumcision improves hygiene 🞏 🞏 🞏

Circumcision reduces sensitivity 🞏 🞏 🞏

# 20. What do you believe your current HIV status is?

HIV positive 🞏 HIV negative 🞏 Don’t know 🞏

# 21. When was your most recent HIV test?

In the last 6 months 🞏

Between 6 months and 1 year ago 🞏

Between 1 and 5 years ago 🞏

Over 5 years ago 🞏

Never had an HIV test 🞏

**If never, go to question 23 🡻**

# 22. What was the result of your last HIV test?

HIV positive 🞏 HIV negative 🞏 Don’t know 🞏

**23. How much do you agree with the following statements?**

**I am less worried about HIV infection now that treatments have improved**

strongly disagree disagree agree strongly agree

🞏 🞏 🞏 🞏

**I believe that new drug therapies make people with HIV less infectious**

strongly disagree disagree agree strongly agree

🞏 🞏 🞏 🞏

**P.T.O**
